# Factors motivating intent to leave amongst radiographers employed by public tertiary hospitals in the Gauteng Province, South Africa

**DOI:** 10.4314/ahs.v22i3.72

**Published:** 2022-09

**Authors:** Maureen Nokuthula Sibiya, Thandokuhle Emmanuel Khoza, Busisiwe Pauline Nkosi

**Affiliations:** 1 Deputy Vice Chancellor of Teaching and Learning, Durban University of Technology Gate 1, Steve Biko Campus, 11 Steve Biko Road, Durban 4001; 2 Department of Radiography, Gate 6, Ritson Campus, 11 Steve Biko Road, Durban 4001; 3 Head of Department of Radiography, Gate 6, Ritson Campus, 11 Steve Biko Road, Durban 4001

**Keywords:** Intent to leave, radiographers, public tertiary hospitals

## Abstract

**Background:**

The elements of job satisfaction can be categorized into intrinsic and extrinsic factors. The presence of a higher level of intrinsic factors will result in increased motivation amongst employees, whilst extrinsic factors will result in job dissatisfaction. Decreased job satisfaction levels amongst healthcare professionals are known to create an intent to leave. Hence the need to explore these factors amongst radiographers employed by tertiary hospitals in the Gauteng province of South Africa.

**Objective:**

To determine the influence of intrinsic and extrinsic factors of job satisfaction on intent to leave amongst radiographers employed by public tertiary hospitals in the Gauteng province.

**Methods:**

A quantitative cross-sectional survey guided the study, and a self-administered questionnaire was used to collect data. The sampling technique used for this study was disproportional stratified sampling.

**Results:**

The study had a response rate of 62%. A significant number of the participants (50%) were between the ages of 21–33 years. Also, worth noting that 51% of the participants were newly qualified, 28% were employed for a period of 10–20 years and only 20% were employed for a period greater than 20 years. Diagnostic radiography had the most number of participants at 55%, followed by radiation therapist at 24%, nuclear medicine radiographers at 13%, mammography radiographers at 5% and only 3% were sonographers. Pearson's correlation showed a significant negative correlation with the following extrinsic factors: supervision, r= -.344, p=.000; satisfaction with PMDS, r=-.302, p=.000; human resources processes, r=-.249, p=.001; infrastructure, r=-.236, p=.001; the OSD policy, r=-.233, p=.002; satisfaction with remuneration, r=-.202, p=.006; satisfaction with CPD activities, r=-.201, p=.007; and satisfaction with equipment, r=-.163, p=.029.

**Conclusion:**

Both intrinsic and extrinsic factors are associated with an intent to leave amongst radiographers employed by public tertiary hospitals in the Gauteng province.

## Introduction

Job satisfaction is a positive emotional state resulting from recognition of an individual's work.^1^ It is also considered to be an aggregate of feelings experienced by an individual related to their job, and the attitude arising when those feelings are well-balanced.^2^ Job satisfaction can also be viewed holistically, which relates to general satisfaction, or it can be viewed dimensionally, which relates to specific aspects of the job.^3^ Hence, job satisfaction has been identified as one of the key factors in retaining healthcare professionals.^4^

The elements that influence job satisfaction can be categorized into intrinsic and extrinsic factors.5 Hertzberg's Theory suggests that intrinsic factors (motivators) result in job satisfaction, whereas extrinsic factors (hygiene) result in job dissatisfaction. The presence of motivating factors may encourage employees to work harder whereas the absence of hygiene factors may result in less effort being expended by employees on their duties.^5^ Job satisfaction and motivation are directly linked to turnover or intention to leave amongst healthcare professionals.^5^. Intrinsic factors are recognition; achievement; advancement; the nature of the work undertaken; and responsibility. Motivators such as recognition and achievement may result in more productive and committed employees. Extrinsic factors are pay, company policies, relationships with supervisors, working conditions and feelings associated with a lack of status or security.^6^

According to statistics released by the Department of Health, South Africa, there is a mal-distribution of radiographers between the public and private sectors.^7^This mal-distribution of radiographers between the public and private sectors in South Africa could be caused by a combination of extrinsic and intrinsic factors of job satisfaction. However, it is worth noting that the private sector is better funded by cash paying patients and medical aid contributions, which makes it easier to pay higher salaries to their employees,^8^ thus leading to an increased retention of its healthcare professionals.

In the public sector, a Performance Management and Development System (PMDS) was introduced in 2001 as part of extrinsic company policies. This policy aimed to plan, manage and improve employee performance.^9^The policy was later (2010) lined to the Occupational Specific Dispensation (OSD). The objectives of the OSD were to introduce career pathing based on competencies, experience and performance. To this end, there have not been any studies conducted amongst radiographers to determine the influence of these policies on their job satisfaction, even though there have been reports of disgruntlement from other healthcare professionals concerning these policies.^10^

Therefore, this study aimed to explore the intent to leave amongst radiographers employed by public tertiary hospitals in the Gauteng province, due to decreased levels job satisfaction. It is assumed that radiographers voluntarily vacate their posts in the public sector for the private sector.

## Methodology

### Study design

A quantitative cross-sectional survey was used to determine the impact of motivating factors on intent to leave for radiographers employed by public tertiary hospitals in the Gauteng Province, South Africa. However, it is worth noting that the results presented in this paper are part of a larger exploratory sequential mixed methods study that was conducted amongst radiographers. This paper will only focus on the quantitative results.

### Ethical approval

Ethical clearance to conduct the study was obtained from the Durban University of Technology, IREC 115/18. The gatekeeper's permission was obtained from the Gauteng Department of Health GP201808_044. At the hospital level, the study was approved by the heads of Radiology, and the Chief Operating Officer signed off the permission letter. In one of the hospitals, a teaching hospital at the University of Pretoria, special ethical clearance, Ethic Reference 643/2018, had to be obtained from the University. The questionnaire included a cover letter explaining the study's objectives. If potential respondents completed the questionnaire, they were deemed to have consented to participating in the study.

### Sampling technique

A stratified random sampling technique was used in this study, specifically disproportional stratified sampling. The reason for this type of technique was the expected unequal distribution of participants across the different professions within radiography. Diagnostic radiographers were expected to constitute the majority of participants, whilst ultra-sonographers and mammography radiographers were expected to be the minority. For the purposes of this study, the term ‘radiographer’ describes the five disciplines within radiography, namely diagnostic radiographers, radiation therapists, nuclear medicine radiographers, ultra-sonographers and mammography radiographers. At the time of data collection, a population of N=292 were employed by the four public tertiary hospitals.

### Data collection

The questionnaires were hand delivered and collected by the researcher from February 2019 – May 2019. Potential respondents were given the questionnaire during departmental meetings or at their work-stations. They were given a week to complete and return the questionnaires, which were collected by the researcher at the end of the working week. The same process was repeated for each of the identified hospitals.

### Data collection tool

The results of Phase One, which was focus group interviews, individual interviews and an extensive literature review, were used to develop the questionnaire. The themes that emerged in the qualitative study were the influence of government policies on job satisfaction; the lack of career pathing; poor remuneration; and poor working conditions and human resources processes. It is worth noting that the methodological process of Phase One will not be discussed for the purposes of this paper. In developing the questionnaire, the researcher had to ensure that the questions were short and simple, and avoided double-barrelled, leading, negatively phrased or ambiguous questions. The self-developed and self-administered questionnaire had 48 statements and used a 5-point Likert scale to rate each statement ranging from 1=strongly and 5=strongly disagree. The questionnaire was divided into three sections, where section A was used to ascertain the demographics of the respondents. The following were the demographics that respondents had to indicate: gender; age; race; marital status; experience; position, and radiography discipline.

Section B the conceptualization of job satisfaction by radiographers, categorized into intrinsic and extrinsic factors of job satisfaction. The intrinsic factor that the study tested was career pathing, with sub-categories of rotation in specialized areas; academic growth; continued professional development; and promotion. In addition, four extrinsic factors were tested, namely: government policies (occupational specific dispensation-OSD; the Employment Equity Act-EEA; and the performance management development system- PMDS); unsatisfactory remuneration (poor salaries, overtime and bargaining councils); working conditions (support from management, workload and staff shortages, physical safety and equipment); and the Human Resources department (accessibility, benefits and posts). Both intrinsic and extrinsic factors were tested against the intent to leave amongst radiographers. Section C was open-ended to allow participants to express any additional views that might have been missed by the researcher.

A pilot study was conducted on seven radiographers (four diagnostic radiographers, two radiation therapists and one mammography radiographer). This resulted in two questions being simplified, six questions added and two questions removed.

### Statistical methods and analysis

A total of 182 questionnaires were distributed and collected out of a possible 292 radiographers employed at the four tertiary hospitals in the Gauteng province, which equates to a response rate of 62%. The questionnaires were coded and captured onto an excel spreadsheet, thereafter, it was uploaded onto an SPPS software version 24.

There was an unequal distribution in the age of participants where 50% were between the ages of 21–33, 31% were between the ages of 34–49 and only 19% were above the age of 50. With regard to race, 68% of the participants were Black, while 17% were Whites, 7.7% were Indians and lastly 5.5% were Coloureds. The results further showed that 49% of the participants were single and 42% were married, while only 6% were separated or widowed. A total of 51% were newly qualified radiographers, while 28% were employed for a period of 10–20 years and only 20% were employed for a period greater than 20 years. Lastly, 55% of participants were diagnostic radiographers, while 24% were radiation therapists; 13% were nuclear medicine radiographers; 5% were mammography radiographers; and only 3% were sonographers.

To reduce the number of statements, Confirmatory Analysis and a Cronbach's alpha coefficient were calculated for each statement. Two types of factor analysis can be performed, namely exploratory factor analysis and confirmatory factor analysis. For the purposes of this study, confirmatory factor analysis was used to confirm a specific hypothesis related to the structure of the set of statements that were grouped together. Literature sources differ on when and how factor analysis should be used. Some recommend that factor analysis should only be used in larger groups because the correlation coefficients in small variables are less reliable .^11^ Large sample sizes are considered to have a minimum of 300 cases, while a small sample size has a minimum of 150 cases. However, some authors suggest that researchers should focus on the ratio of participants per item, such as a ratio of 10 cases per item.^12^ In this study, some items were grouped in relation to their similarities.

**Figure 1:**
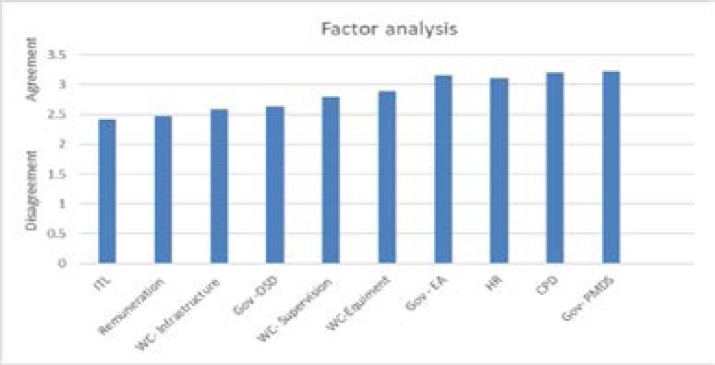
Factor analysis on level of agreement

A total of 10 factors emerged after factor analysis was performed, and these were tested for internal consistency. The results showed an acceptable level of the Cronbach's alpha coefficient: intention to leave .857; government's policy on PMDS .831; government's policy on OSD .807; government's policy on EEA .809; favourable working conditions provided by supervision .808; favourable working conditions provided by equipment .825; favourable working conditions provided by infrastructure .677; CPD activity .793; and human resources department .684. A one-sample t-test was performed to determine the level of agreement or disagreement for the ten factors that emerged after factor analysis, as shown in Figure 1. The results indicate that there was a significant disagreement (M=2.6363) that the OSD policy was fairly implemented, t (181) = -5.665, p<.0005. Moreover, there was a significant disagreement that supervision creates favourable working conditions, t (181) = -5.219, p<.005; there was a significant disagreement (M=2.5889) that the infrastructure creates favourable working conditions; and there was a significant disagreement that the remuneration was fair, t (180) = -6.268, p< .0005. The following statements had a significant level of agreement: there was a significant agreement that the EEA is fairly implemented, t (181) = 2.312, p=.022; there was a significant agreement (M=3.3048) that the PMDS tool is fairly used t (181) =2.840, p=.05; and there was a significant agreement (M=3.2019) that departments had relevant CPD activities, t (179) = 2.659, p<.009. Some of the factors had no significant level of agreement or disagreement, namely the efficiency of the human resources department and that equipment provided favourable working conditions.

A Pearson's correlation was performed to determine the correlation between intention to leave (ITL) and the nine factors that emerged during Confirmatory Factor Analysis, namely government's policy on PMDS; government's policy on OSD; government's policy on EEA; favourable working conditions provided by supervision; favourable working conditions provided by equipment; favourable working conditions provided by infrastructure; CPD activity; and human resources department.

The results are presented in their order of significance, with supervision being strongly correlated with intention to leave as demonstrated in Table 1.

**Table 1:** Pearson's correlation on intent to leave

	Supervision	PM DS	HR	Infrastructure	OSD policy	Remuneration	CPD activity	Equipment	EEA policy
ITL Pearson Correlation	-.344	-.302	-.249	-.236	-.233	-.202	-.202	-.163	-.086

## Discussion

The study aimed to explore the link between intrinsic and extrinsic factors and intent to leave amongst radiographers employed by public tertiary hospitals. Career pathing, or the lack there of, was the only intrinsic factor explored in this study. Previous studies have reported that organisations could enhance organisational commitment amongst employees by helping them to achieve their career goals, acquiring new skills and offering financial incentives.^13^ However, the results of the one-sample t-test showed a significant disagreement that a radiographer's postgraduate qualification was recognized in their remuneration. Similarly, there was no financial recognition for acquiring new skills, such as training in Computed Tomography (CT) and Magnetic Resonance Imaging (MRI) for diagnostic radiographers, while it was the department that focuses treatment planning for radiation therapists. The extrinsic factors were also significantly linked to intent to leave amongst radiographers. In the three government policies tested in the study, two were found to have a significant correlation with ITL, namely the OSD and PMDS policies. The negative connotation associated with the OSD policy was that of salary stagnation and the inconsistent application of the policy across the disciplines, which has been previously reported by other authors.^11^,^14^,,^15^ The results showed that radiation therapists were the least satisfied with the OSD policy's implementation process, whereas dissatisfaction with the PMDS policy was consistent throughout the different disciplines, that being the unfair rating of subordinates by their supervisors. It has been previously reported that supervisors lacked the required skills and expertise to rate their subordinates effectively.^16^ The PMDS ratings could result in radiographers being on one salary scale for a lengthy period, hence its significant correlation with intention to leave. Furthermore, the OSD and the PMDS policy are interlinked in the application of accelerated grade progression. Therefore, it is no surprise that remuneration was significantly correlated with the intent to leave amongst radiographers.

Furthermore, the OSD Policy has failed to recognize radiographers employed in a speciality without dual qualification, thus negatively affecting job satisfaction and increasing intent to leave. Previously, radiographers would only be allowed to study sonography, radiation therapy and nuclear medicine after the have completed an undergraduate diploma in diagnostic radiography.^17^ However, due to staff shortages and other challenges, students were accepted into these qualifications without an undergraduate qualification in diagnostic radiography. In addition, the OSD Policy did not created supervisory posts for a speciality within radiography (sonography, radiation therapy and nuclear medicine), thus limiting career growth in terms of promotion. Furthermore, the accelerated grade progression introduced by the OSD Policy is directly linked to the PMDS rating received by an individual. Accelerated grade progression means that radiographers who receive a rating of 4–5 in their PMDS ratings qualify to move onto the next salary grade. Should they fail to obtain these ratings, then they would remain on one salary grade for a period of 10 years. However, participants have cited that they were not fairly rated by managers, which resulted in salary stagnation caused by being in one position for a period of 10 years.

The leading factor that was negatively correlated with intent to leave is that of supervisor-relationship as demonstrated by the Pearson's correlation on intent to leave. Newly qualified radiographers showed the least satisfaction with their supervisor-relationship. The majority of participants in this age category are considered to be Generation Y, also known as millennials. This is the generation born between 1981 and 1994/6. These individuals tend to build multiple careers and have more than one job at a time, hence inter-organizational transfer would be easy for them.^18^ It is also worth noting that there was a difference in the level of satisfaction with the type of supervision offered across the four tertiary hospitals included in the study. Radiographers employed in tertiary hospital Number Two showed the greatest satisfaction with their supervisor-relationship, whereas tertiary hospital Number Three showed the least satisfaction.

The infrastructure, which was related to a functional state of imaging equipment, was also deemed to be a source of extrinsic factors related to job dissatisfaction. This is despite the introduction of the National Core Standards by the Department of Health in 2011. Part of the objectives of this policy was to ensure that equipment is well maintained, in a fully functional state, complies with regulations and when needed, it is upgraded or replaced. The findings were consistent with previous studies where the functional state of equipment was linked to lower levels of job satisfaction and greater intent to leave.^3^,^19^

## Limitations

The questionnaire was only distributed to radiographers who were on duty at the time of data collection, which could have introduced response bias.

Participants could have exaggerated their responses with the hope that the study would effect immediate changes to their work situation.

The study was only conducted in the public sector; hence the findings cannot be generalized to the private sector.

## Conclusion

The results of this study demonstrated that the there was a strong correlation between extrinsic factors of job satisfaction and intent to leave. The four extrinsic factors that directly linked with intent to leave were government policies; working conditions; infrastructure and supervision; and the efficiency of the human resources department. It is worth noting that the majority of these factors are caused by human elements and therefore could be addressed with proper implementation. This challenges the employer (government) to develop monitoring mechanisms to ensure that their policies are properly and fairly implemented and secondly, to ensure that infrastructure is upgraded and maintained at acceptable levels. It is hoped that these measures might increase the retention rate of radiographers employed by public tertiary hospitals in the Gauteng province.
